# Web-based support for individuals with type 2 diabetes - a feasibility study

**DOI:** 10.1186/s12913-021-06707-7

**Published:** 2021-07-22

**Authors:** Marina Taloyan, Meybod Kia, Fahimeh Lamian, Magnus Peterson, Elisabeth Rydwik

**Affiliations:** 1 Academic Primary Care Center, Region Stockholm, Stockholm, Sweden; 2 Dept of Neurobiology Care Sciences and Society, Division of Family Medicine and Primary Care, Karolinska Institutet, Alfred Nobels allé 23, SE-14183 Huddinge, Sweden; 3 Capio Solna Primary Health Care Center, Region Stockholm Stockholm, Sweden; 4 Capio Väsby Primary Health Care Center, Region Stockholm Stockholm, Sweden; 5 Department of Public Health and Caring Sciences, Section of General Medicine, Uppsala University, Uppsala, Sweden; 6 Research and Development Unit for the Elderly, FOU nu, Region Stockholm Stockholm, Sweden; 7 Departement of Neurobiology Care Sciences and Society, Division of Physiotherapy, Karolinska Institutet, Solna, Sweden; 8 Karolinska University Hospital, Women’s Health and Allied Health Professional Theme, Medical Unit Occupational therapy and Physiotherapy, Solna, Sweden

**Keywords:** Type 2 diabetes, Web-based support, Feasibility

## Abstract

**Background:**

Self-care is one of the cornerstones in the treatment of type 2 diabetes. Patients with type 2 diabetes struggle to maintain acceptable levels of blood sugar, blood pressure and lipids, the fundamental for the prevention of macro- and microvascular as well as neuropathic complications. The primary aim of the study was to evaluate the feasibility and describe patients’ and caregivers’ experiences of using the web- and smartphone-based system Triabetes. The secondary aim was to investigate if the use of the system could improve patients’ clinical outcomes.

**Methods:**

Feasibility was assessed with describing recruitment rate and the participant´s views of using the system. Laboratory and anthropometry data were also collected.

**Results:**

The study showed that recruitment of patients to participate in the intervention was limited and compliance to the study protocol was low. A majority of the patients stated that the system was easy to get an overview of and that the system motivated them and made it easier and fun to handle lifestyle habits. A secondary finding of the study was that there was a significant lowering of LDL values.

**Conclusions:**

Feasibility in terms of recruitment rate was low. The participants agreed that the application overall was useful but suggested several improvements. Summarized lessons learned from this study are following: (1) we need more knowledge about what motivates a person to use a digital tool for a longer period of time; (2) the tool must be easy and less time consuming to use; (3) the technical structure needs to be improved and automatic recording of data must be improved.

**Supplementary Information:**

The online version contains supplementary material available at 10.1186/s12913-021-06707-7.

## Background

Diabetes type 2 is a chronic health condition increasing globally [1]. The disease can lead to several serious complications from the eyes, kidneys and nerves and doubles the risk of cardiovascular disease compared to people without diabetes [2–4]. The majority of people with type 2 diabetes are overweight, due to both genetic and lifestyle factors [3]. In addition, prediabetes is a growing issue as well, with 10 % of the adult population in Europe estimated to be afflicted [1]. Without lifestyle changes, up to 30 % of these could develop diabetes within 5 years [4]. Over 2 million deaths annually are related to high blood glucose alone [1].

For people with type 2 diabetes, proper maintenance of blood sugar, blood pressure and lipids are fundamental for the prevention of macro- and microvascular as well as neuropathic complications [5]. Many patients struggle with maintaining acceptable levels, and the consequences are low quality of life, early mortality and high societal costs due to reduced working capacity and high number of consultations in both primary health care and hospitals. Type 2 diabetes is no longer an adult-onset disease. The number of young individuals with type 2 diabetes is increasing [1]. Younger ages have been shown to be associated with poorer HbA1c control and increasing cardiovascular risk factors [6]. Lifestyle changes are effective in delaying the onset of type 2 diabetes and diabetes complications.

Self-care is one of the cornerstones in the treatment of type 2 diabetes [7, 8]. Even when following recommendations of regular check-ups within health care once or twice a year [9, 10], the rest of the year people with diabetes are depending on self-care involving diet, physical activity, and restraint tobacco use and alcohol intake alone or in combination with pharmacological treatment (oral drugs and insulin). Lowering blood glucose is crucial for reducing damaging effects on blood vessels.

Already in 2006, an RCT study in Maryland showed that using cell phone based software for real-time feedback on patients, improved HbA1c significantly [11]. Recent reviews have shown that web-based support and telemedicine can have an effect on HbA1c and systolic blood pressure but show conflicting result on other outcomes [12, 13]. Another review, investigating patients satisfaction with telemedicine in diabetes management, showed that individualized telemedicine strategies together with sufficient technical support as well as support from the physician could improve usability and sustainability [14]. In Sweden 2016, 82 % of the population were estimated to be daily users of the internet [15]. In 2019 the prevalence of daily internet users had increased to 91 % [16]. Digitalization of health care is a priority issue in many countries, where patients have access to their own medical record on the web. Telemedicine has the potential to address many key challenges of providing health care for populations dispersed in large geographic areas [17, 18]. This has a great potential not only for rural and remote areas but also populations dispersed in an urban community. Med-Tech companies offer a large and growing number of digital solutions and tools for health care. In the context of the covid-19 pandemic, the use of various digital tools has accelerated. However, few tools are still implemented in daily care and future studies will need to explore this relatively new phenomenon in healthcare. Worldwide, the use of digital services is steadily increasing, and more and more people are frequently using digital tools for everyday activities in their lives.

Triabetes is one of many existing digital tools for patients with diabetes (see description below). Feasibility of an intervention with Triabetes might fill the gap of knowledge regarding implementation of new digital tools within primary health care. If some patients could manage their own care through tools such as Triabetes, caregivers in primary care can focus more on other patients that need more support. Reporting of this pilot trial is presented according to the guidelines of Consolidated Standards of Reporting Trials (CONSORT) [19].

The primary aim of the study was to evaluate the feasibility and describe patients’ and caregivers’ experiences of using the web- and smartphone-based system Triabetes. The secondary aim was to investigate if the use of the system could improve patients’ clinical outcomes.

## Methods

### Study design

The current study is a feasibility study exploring both process feasibility and scientific feasibility.

### Recruitment of participants

The study population consisted of health care providers and enlisted patients at the Jakobsberg’s Academic Primary Health Care Center (APHC), Stockholm, Sweden. The catchment area is suburban and consists of mixed ethnic and socioeconomic groups living in communal housing as well as own homes.

#### Health care providers

General practitioners (GP:s) and diabetes nurses (both groups referred to as health care providers below) at the APHC were asked for participation in the study. At the center, there are 11 GPs and 2 diabetes nurses employed, and there are on average 65 listed patients with type 2 diabetes per GP. Four GPs and one diabetes nurse consented to participate.

#### Participants

An information letter about the study was sent to patients with type 2 diabetes or prediabetes who were listed at the center asking for interest to participate in the study. The letters were followed up by telephone calls by the principal investigator. Patients with type 2 diabetes with HbA1c < 48 mmol/mol and BMI < 18.5 were excluded. Exclusion criteria for both prediabetes and diabetes groups were age under 18 years; did not understand or could use smartphone technology and/or had no computer and internet access or could not read the Swedish language.

#### Description of the intervention Triabetes

Triabetes is based on two parts: a smartphone-based application for the patients and a web system for the health care providers with focus on monitoring, coaching and decision-making support for patients with diabetes towards the goal of achieving glycemic and metabolic control through lifestyle changes. The goal of the tool is to strengthen the patient’s autonomy by presentation of simple diagrams of physical activities, weight loss, eating habits and glycemic control. Each patient sets individual goals together with the health care providers regarding blood glucose, weight, diet and physical activity. The web- and application-based system gives direct feedback regarding number of steps and other physical activities. Patients can register food intake manually and get feedback based on the Swedish National Food Agency’s database. The system also includes reminders of time for medication and physical activity.

Care providers have an alarm and information system and can follow the patient’s history and health condition over time through the web-based system for setting individual goals and planning of activities. It is possible to visualize and see acute situations and negative trends through an overall analysis of data for all patients and to see who needs more focus and support during certain periods.

### Procedure

 To those who consented to participate, time for an appointment with the GP was sent by letter together with written informed consent and instructions about visiting the laboratory before the GP visit. After the GP visit, the GP contacted the diabetes nurse who in turn called the participant to schedule a meeting. At follow-up, an appointment to the GP were sent by letter together with a questionnaire about usability and instructions about visiting the laboratory before the follow-up GP visit.

#### Intervention

The intervention consisted of using the Triabetes application/web system for health care providers during working time and for patients at home. The GPs’ were given education on the system at one half day and the nurse at two half days (the nurse was the one who instructed the patients).

The patients were instructed by the nurse at the baseline visit, regarding how the application and the web system worked, how they recorded data and how they could track their results through the web application. The healthcare providers recorded laboratory data, medications and the goals in the Triabetes web system. They also identified patients who needed extra support. This could for example be due to baseline laboratory reference values such as HbA1c and β-glucose or the goals set individually for each patient such as losing weight. The GP set the goals together with the patient, which was then followed up by the diabetes nurse by phone or visits depending on the patient’s wishes and need for support. For the patient, the intervention including registration of eating habits and physical activities and lasted for 6 months.

### Primary outcome measures (process feasibility)

Presentation of the process feasibility measures in this study includes recruitment procedure, usability of the intervention and acceptance among both patients and caregivers [20].

The recruitment procedure and rate as well as dropout and the amount of missing data was documented. To evaluate usability of the application/system the healthcare providers and patients answered a questionnaire about how they experienced the use of the system and how much they used it. The questionnaire developed for this study is provided as Additional Files 1 and 2. The experience of the system was evaluated by 24 statements for the participants and 25 for the health care providers with a 4-graded scale from “Strongly disagree” to “Strongly agree”. At the end of the questionnaire, there were open-ended questions about advantages and disadvantages with the application/system as well as suggestions for improvements.

### Secondary outcomes (scientific feasibility)

The secondary outcomes relates to scientific feasibility, as described by Thabane et al. 2010, including for example estimation of preliminary treatment effects [20]. The following health data variables were collected at baseline and after 6 months:

- Laboratory (fasting): HbA1c, lipids (High-density
- Lipoprotein (HDL)/Low-density Lipoprotein (LDL)), β-glucose, Total Cholesterol, Triglycerides.
- Anthropometry: Height and weight (base for calculation of BMI) and waist circumference.

### Statistical analyses

Descriptive data are presented with number or median and interquartile range due to the small sample size. The 4-graded scale in the usability questionnaires was dichotomized. Differences between baseline and follow-up regarding health variables were calculated with Wilcoxon Sign test. The open-ended questions were analyzed with content analysis and are presented as themes.

## Results

### Process feasibility

#### Recruitment

**Fig. 1:**
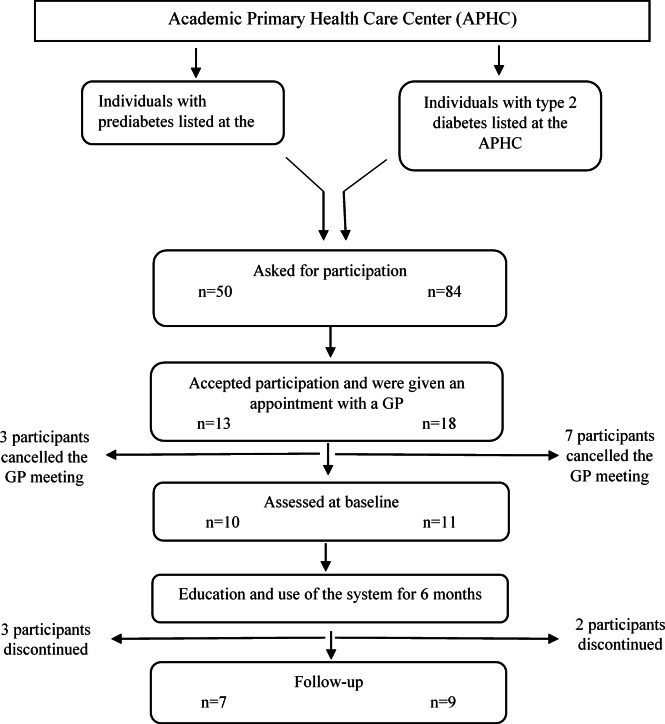
Flow chart describing the procedure of data collection

Letters about the study were sent to patients that were listed with the four GPs’ and fulfilled inclusion criteria (n = 138). In the letter, the patients were asked to contact the APHC if they wanted to participate in the study. Only three participants contacted the APHC and showed interest to participate. The letters were therefore followed-up by telephone calls by the principal investigator. Of the 135 contacted by phone, 68 were not reachable and/or did not return the telephone message, 39 declined participation and 28 consented to participate. In total, 31 patients consented to participate, 16 continued participation and were assessed at follow-up. Median age was 60 years (Q1-Q3 54–71), 16 men and 15 women, see Fig. 1.

#### Usability

Fifteen of the remaining 16 participants answered the questionnaire about usability, however two of those stated that they never had logged into the system and did therefore not answer the following questions. One participant did not answer the questions about how much he/she had used the system. A majority of the participants logged in several times /week and nine participants stated that they would consider using the system for support during a specific and defined period in the future.

Of the health care providers, two of the GP’s and the nurse answered the questionnaire about the system. The nurse estimated that it took on average 1–10 min to record a patient the first time and the GPs’ that it took 10–20 min. During follow-up meetings, the estimated time was 5–10 min.

A majority of the participating patients stated that the system was easy to get an overview of, to use, and understand and that they recommended the system. However, only half of the participant agreed that it was easy to record data and that it worked as expected. A majority also stated that the system motivated them and made it easier and fun to handle lifestyle habits. In addition, 11 of 13 participants definitely recommended the system (see Table 1).

**+ 1:** The participating patients responds to the usability of the system (*n* = 13)

What do you think of Triabetes system as a whole:	Strongly disagree / disagree (n)	Partly agree / Strongly agree (n)
Easy to learn	2	11
Easy to log in	2	11
Easy to get an overview	1	12
Easy to navigate	3	10
Easy to read information	2	11
Easy to understand information	0	13
Easy to record	6	7
Helps me to do what I planned	6	7
Works as expected	6	7
Support me to eat according to goals	5	8
Support me to exercise according to goals	4	9
Easier to handle my lifestyle habits	3	10
More fun to handle my lifestyle habits	4	9
Motivates me to exercise more and eat correct	3	10
Better overview of my health	3	10
Communication with health care has improved	4	8
Easier to follow-up and evaluate effects of treatment	5	8
My knowledge and my way of handling lifestyle habits has improved	4	9
Definitely recommend the system	2	11
Esthetical appealing	4	9
Now I need less contact with health care	7	5
It seems safe	3	10
The system sometimes hatches (“bugs”)	5	8
Easy to do wrong	6	7

The health care providers also agreed that it was easy to use and understand. However, none of them agreed that it saved time, made their work easier or increased their knowledge on how to treat the patients (see Table 2).

**Table 2:** The health care providers respond to the usability of the system (*n* = 3)

What do you think of Triabetes system as a whole:	Strongly disagree / disagree (n)	Partly agree / Strongly agree (n)
Easy to learn	0	3
Easy to log in	0	3
Easy to get an overview	0	3
Easy to navigate	1	2
Easy to read information	1	2
Easy to understand information	0	3
Easy to record	2	0
Helps me to do what I planned	1	2
Works as expected	2	1
Helps me in my work	1	2
The system sometimes hatches (“bugs”)	0	3
Easy to do wrong	2	1
My work gets easier	3	0
My work gets more satisfying	2	1
My work gets more fun	1	2
It motivates me to do a good job	2	1
Communication with patients have increased	0	3
Easier to follow-up and evaluate treatment	1	2
My knowledge and my way of treating patients has improved	3	0
Esthetical appealing	1	2
It seems safe	1	2
It saves time	3	0
I get a better overview of the patient’s health	0	3
I have someone to ask when I don’t understand	0	3
I get support from the company when I need	1	2

### Result of open answers

The questionnaire included four questions for free text. Patients responded in written form regarding disadvantages and benefits of using the app/web support, development or and improvement suggestions and other comments. Analysis of these written comments/free text in the questionnaire resulted in three themes: *benefits, weaknesses and suggestions for improvement*. Several categories emerged under the theme:

*Benefit*: it “forced” regularity, motivated to change eating habits, showed variations through visualization/graphs, helped to control own habits, follow up and see results.

*Weaknesses* with the application were: too time demanding, lack of possibility to go back to registered data, weak technical structure and limited response alternatives for food registration.

*Suggestions for improvement* were: registration of drug intake and goal for treatment, reward with happy signals and to inform health providers when data is registered.

### Scientific feasibility

#### Preliminary treatment effects

The results of the laboratory data and anthropometry are shown in Table 3. On the median level there were no differences between baseline and follow-up except for LDL (see Table 3). The distribution of weight varied between 62 and 130 kg at baseline, 4 participants declined, 1 increased and the rest were stable at follow-up.

**Table 3:** Results of health variables at baseline and follow-up

Variables	Baseline md (Q1-Q3)	Follow-up md (Q1-Q3)
HbA1C (IFCC) (*n* = 14)	43.5 (37.75–56.25)	43.5 (36.75–51.25)
β-glucose (mmol/L) (*n* = 14)	5.65 (5.08–9.08)	6.45 (5.60–7.20)
Cholesterol (mmol/L) (*n* = 12)	4.90 (4.53–5.28)	5.15 (4.73–5.50)
Triglyceride (mmol/L) (*n* = 12)	1.30 (0.93–1.70)	1.75 (1.13–2.08)
HDL (mmol/L) (*n* = 12)	1.40 (1.20–1.60)	1.35 (1.20–1.50)
LDL (mmol/L) (*n* = 12)	3.10 (2.88–3.78)	2.85 (2.45–3.35) *
Weight (kg) (*n* = 13)	88.0 (75.1–101.0)	87.5 (71.75–97.00)
BMI (*n* = 13)	29.05 (27.78–31.90)	28.70 (26.58–31.58)

*p < 0.05

## Discussion

The main results of the study show that recruitment of patients to participate in the intervention was limited and compliance to the study protocol was low. A secondary finding of the study was that the only significant treatment effect was a lowering of LDL values.

A number of studies describe factors explaining the patient’s participation in interventions. In a large number of the studies, findings suggest that families with the largest needs participate in interventions to a lesser extent. Demographic and socioeconomic factors as well as belonging to minority groups have been associated with both initial engagement and longer attendance in interventions [21, 22].

Previous studies evaluating effects and satisfaction with telemedicine solution in people with type 2 diabetes has not reported the recruitment rate, most often only stated that they included a convenient sample [23, 24]. However, two studies reported that 46–47 % of eligible patients were included compared to 22 % in the current study [25, 26]. One explanation for the low recruitment rate in our study could be that the catchment area of the APHC consists of a population with varying socioeconomic status and a diverse ethnic background with a large group with origin from the Middle East, in which negative consequences of emigration on health and language barriers can be possible explanations of declining participation [27]. Additional explanations may be related to multi-morbidity and lack of interest or hope in improving individual health by a digital application. These possible explanations for limited participation and compliance in the study are in bright contrast to the importance of finding novel ways to motivate non-European immigrants to become involved in studies and interventions that may improve their health. In fact, those born outside Europe have 3–4 times higher prevalence of the diabetes and obesity, poorer self-rated health and 10 years earlier onset of diabetes than native Swedish population [28, 29], and thus should be in focus of interventions.

 Another interesting finding was that of those who had given verbal consent to participate, one third cancelled the first meeting with the GP. There could be several reasons for this. In the letter about the scheduled meeting, detailed written information about the study was included and maybe they realized the effort that they might have to put into participating. Another factor could be that good communication between patient and doctor increase patient’s compliance, which can lead to better health. Patient’s compliance might be depending on several factors. According to multi-center study done in Belgium, UK, Italy and The Netherlands are perspectives in communication styles and physicians’ competency important for doctor-patient communication. Patient’s educational level can also influence communication between doctors and patients. Lower educated patients preferred emotional aspects while middle and high educated patients focused on task/problem-oriented areas of communication [30].

Another 16 % in our study discontinued the intervention and cancelled their follow-up meeting with the GP. Attrition rates (8, 11, 21 %) has been reported in previous studies [26, 31, 32] and are similar to our study. However, the length of the intervention varied between 3 and 12 months in the different studies. Deeper understanding of reasons why patients decided not to participate or drop out of these types of intervention is warranted. Future studies on new technical innovation should include qualitative methods regarding experiences and perceptions of those who refuse to participate or drop-out during the intervention.

A systematic review has reported that satisfaction with mobile applications is high [33]. In our study, the participants reported that the application increased their motivation to handle life style habits and similar results have been reported in studies [31, 34]. However, several participants also reported that they disagreed to the statement that it was “easy to record” or “helped them to do what they planned”. Open answers also showed that the system was too time demanding, had a weak technical structure and that there were limited response alternatives for food registration. It is difficult to compare these results with other studies since it is not possible to determine in what way the systems/application differed or were similar. However, several studies have reported that many participants have stated that it’s time consuming to use the systems and that compliance for using the system declined during the intervention period [31, 34].

Scientific feasibility in this study was evaluated by reporting treatment effects. LDL declined significantly, which is opposite compared to what is reported in other studies [26, 31]. In addition, we could not find a significant decline in HbA1c, which is partly similar to other studies. Two reviews have reported contradictory results in HbA1c [33, 35].

Limitations of the study are that the design and the small sample size in this study do not allow us to draw any conclusions regarding treatment effects, and accordingly these could gain from further evaluation with a larger study sample. Another limitation of the study is that feasibility in terms of recruitment rate was low.

## Conclusions

The results of this study can be concluded as following: (a) feasibility in terms of recruitment rate was low, but participants agreed that the application Triabetes overall was useful; (b) participants requested easier and less time-consuming digital tools; and finally d) using the application Triabetes may results in significant lowering of LDL-values.

Lessons learned from this study can be summarized as follows: (1) effectiveness of digital solutions in health care may be limited by patients’ acceptance and we need more knowledge about what motivates people of different ethnic and socioeconomic backgrounds suffering from diabetes type 2 to use a digital tool for a longer period of time; (2) the tool must be easy and less time consuming to use; (3) the technical structure needs to be improved and automatic recording of data must be improved. In the light of the present pandemic where the work against global public health issues may gain even more from distance-operated technical advancements, it is an important lesson that the effectiveness of modern technologies is only as effective as they are accepted.

## Supplementary Information

**Additional file 1.**

**Additional file 2.**

## Data Availability

Neither data nor materials are publicly available as according to information included in written informed consent that data will be shared and available only with and to the research group in the current study. Unidentified datasets of the current study are available from the corresponding author on reasonable request.

## Abbreviations

APHC: Academic Primary Health Care Center
BMI: Body Mass Index
CONSORT: Consolidated Standards of Reporting Trials
HbA1c: Glycated haemoglobin
HDL: High-density Lipoprotein
GP: General Practitioner
Kg: Kilogram
LDL: Low-density Lipoprotein
Q1-Q3: Interquartile range
UK: United Kingdom
