# Supplementary material for: Web-based support for individuals with type 2 diabetes - a feasibility study
Source: BMC Health Serv Res. 2021 Jul 22;21:721. doi: 10.1186/s12913-021-06707-7 (PMC8295635; doi:10.1186/s12913-021-06707-7)
Supplement: Supplementary file 1 — Additional file 1. [file 12913_2021_6707_MOESM1_ESM.docx]

**Questionnaire on the use of the application/websupport Triabetes**

- The questions have different responce options to choose from. Mark with a cross the option that best matches your opinion
- Try to answer the questions as honestly as possible without thinking too long about each question
- Only one cross per question
- The first part is about the extent to which you have used the system and how far it has taken to enter data. The second part is about the system as a whole and if you feel that it has been a support for you.
- Answer all questions.

**Thank you for your participation!**

How often have you logged in to the app /web support to enter data?

A couple of times a day ☐

Once a day ☐

A couple times a week ☐

One a week ☐

A couple times a month ☐

Once a month ☐

Once a quarter ☐

Never ☐

How long has it taken to enter information about your diet?

<5 min/day ☐

5 min/day ☐

10 min/day ☐

15 min/day ☐

20 min/day ☐

How long has it taken to enter information about exercise activities other than walking?

<5 min/day ☐

5 min/day ☐

10 min/day ☐

15 min/day ☐

20 min/day ☐

I could imagine to use app/ web support

Every day continuously ☐

For a period of time to receive increased support ☐

For a period a couple of times a year ☐

Never ☐

| What do you think of Triabetes system as a whole: | Strongly disagree | Disagree | Partly agree | Strongly agree |
| --- | --- | --- | --- | --- |
| Easy to learn |  |  |  |  |
| Easy to log in |  |  |  |  |
| Easy to get an overview |  |  |  |  |
| Easy to navigate |  |  |  |  |
| Easy to read information |  |  |  |  |
| Easy to understand information |  |  |  |  |
| Easy to record |  |  |  |  |
| Helps me to do what I planned |  |  |  |  |
| Works as expected |  |  |  |  |
| Support me to eat according to goals |  |  |  |  |
| Support me to exercise according to goals |  |  |  |  |
| Easier to handle my life style habits |  |  |  |  |
| More fun to handle my life style habits |  |  |  |  |
| Motivates me to exercise more and eat correct |  |  |  |  |
| Better overview of my health |  |  |  |  |
| Communication with health care has improved |  |  |  |  |
| Easier to follow-up and evaluate effects of treatment |  |  |  |  |
| My knowledge and my way of handling life style habits has improved |  |  |  |  |
| Definitely recommend the system |  |  |  |  |
| Esthetical appealing |  |  |  |  |
| Now I need less contact with health care |  |  |  |  |
| It seems safe |  |  |  |  |
| The system sometimes hatches ("bugs") |  |  |  |  |
| Easy to do wrong |  |  |  |  |

**Write down some disadvantages with the system:**

_________________________________________________________________

_________________________________________________________________

_________________________________________________________________

**Write any development/improvement you would like to see:**

___________________________________________________________________

___________________________________________________________________enk

___________________________________________________________________

**Write some benefits of the system:**

___________________________________________________________________

___________________________________________________________________

___________________________________________________________________

**Other comments:**

___________________________________________________________________

___________________________________________________________________

___________________________________________________________________
