# Supplementary material for: Web-based support for individuals with type 2 diabetes - a feasibility study
Source: BMC Health Serv Res. 2021 Jul 22;21:721. doi: 10.1186/s12913-021-06707-7 (PMC8295635; doi:10.1186/s12913-021-06707-7)
Supplement: Supplementary file 2 — Additional file 2. [file 12913_2021_6707_MOESM2_ESM.docx]

**Questionnaire on the use of the Triabetes system**

- The questions have different responce options to choose from. Mark with a cross the option that best matches your opinion
- Try to answer the questions as honestly as possible without thinking too long about each question
- Only one cross per question
- Answer all questions
- Your profession: nurse ☐ doctor ☐

**Thank you for your participation!**

1. How many times have you used the system in connection with a patient visit:____________________________
2. How often have you logged in to follow a patient’s progress?

Never ☐

Once in six months ☐

Once in quarter ☐

Monthly ☐

A couple of times a month ☐

Once a week ☐

A couple of times a week ☐

Every day ☐

1. How long does it take to enter information about a patient?

5 minutes ☐

10 minutes ☐

15 minutes ☐

20 minutes ☐

>20 minutes ☐

1. How long does it take to go in and follow a patient?

1 minute ☐

2-4 minutes ☐

5-7 minutes ☐

7-10 minutes ☐

>10 minutes ☐

Have not done ☐

| What do you think of Triabetes system as a whole: | Strongly disagree | Disagree | Partly agree | Strongly agree |
| --- | --- | --- | --- | --- |
| Easy to learn |  |  |  |  |
| Easy to log in |  |  |  |  |
| Easy to get an overview |  |  |  |  |
| Easy to navigate |  |  |  |  |
| Easy to read information |  |  |  |  |
| Easy to understand information |  |  |  |  |
| Easy to record |  |  |  |  |
| Helps me to do what I planned |  |  |  |  |
| Works as expected |  |  |  |  |
| Helps me in my work |  |  |  |  |
| The system sometimes hatches ("bugs") |  |  |  |  |
| Easy to do wrong |  |  |  |  |
| My work gets easier |  |  |  |  |
| My work gets more satisfying |  |  |  |  |
| My work gets more fun |  |  |  |  |
| It motivates me to do a good job |  |  |  |  |
| Communication with patients have increased |  |  |  |  |
| Easier to follow-up and evaluate treatment |  |  |  |  |
| My knowledge and my way of treating patients has improved |  |  |  |  |
| Esthetical appealing |  |  |  |  |
| It seems safe |  |  |  |  |
| It saves time |  |  |  |  |
| I get a better overview of the patients health |  |  |  |  |
| I have someone to ask when I don’t understand |  |  |  |  |
| I get support from the company when I need |  |  |  |  |

**Write down some disadvantages with the system:**

___________________________________________________________________

___________________________________________________________________

___________________________________________________________________

**Write any development/improvement you would like to see:**

___________________________________________________________________

___________________________________________________________________

___________________________________________________________________

**Write some benefits of the system:**

___________________________________________________________________

___________________________________________________________________

___________________________________________________________________

**Other comments:**

___________________________________________________________________

___________________________________________________________________

___________________________________________________________________
